# Antibiofilm Activity of Polyamide 11 Modified with Thermally Stable Polymeric Biocide Polyhexamethylene Guanidine 2-Naphtalenesulfonate

**DOI:** 10.3390/ijms20020348

**Published:** 2019-01-16

**Authors:** Olena Moshynets, Jean-François Bardeau, Oksana Tarasyuk, Stanislav Makhno, Tetiana Cherniavska, Oleg Dzhuzha, Geert Potters, Sergiy Rogalsky

**Affiliations:** 1 Institute of Molecular Biology and Genetics of NAS of Ukraine, 03143 Kyiv, Ukraine; 2 Institut des Molécules et Matériaux du Mans, UMR CNRS 6283, Université du Mans, 72085 Le Mans, France; jean-francois.bardeau@univ-lemans.fr; 3 V. P. Kukhar Institute of Bioorganic Chemistry and Petrochemistry of NAS of Ukraine, 02160 Kyiv, Ukraine; oksanatarasyuk@bigmir.net (O.T.); dzhuzha.oleg@gmail.com (O.D.); 4 Chuiko Institute of Surface Chemistry of NAS of Ukraine, 03680 Kyiv, Ukraine; stmax@ukr.net (S.M.); t-cherniavska@ukr.net (T.C.); 5 Antwerp Maritime Academy, Noordkasteel Oost 6, 2030 Antwerp, Belgium; geert.potters@hzs.be; 6 University of Antwerp, Groenenborgerlaan 171, 2020 Antwerp, Belgium

**Keywords:** polyamide 11, antibacterial, polymeric biocide, thermal stability, biofilm

## Abstract

The choice of efficient antimicrobial additives for polyamide resins is very difficult because of their high processing temperatures of up to 300 °C. In this study, a new, thermally stable polymeric biocide, polyhexamethylene guanidine 2-naphtalenesulfonate (PHMG-NS), was synthesised. According to thermogravimetric analysis, PHMG-NS has a thermal degradation point of 357 °C, confirming its potential use in joint melt processing with polyamide resins. Polyamide 11 (PA-11) films containing 5, 7 and 10 wt% of PHMG-NS were prepared by compression molding and subsequently characterised by FTIR spectroscopy. The surface properties were evaluated both by contact angle, and contactless induction. The incorporation of 10 wt% of PHMG-NS into PA-11 films was found to increase the positive surface charge density by almost two orders of magnitude. PA-11/PHMG-NS composites were found to have a thermal decomposition point at about 400 °C. Mechanical testing showed no change of the tensile strength of polyamide films containing PHMG-NS up to 7 wt%. Antibiofilm activity against the opportunistic bacteria *Staphylococcus aureus* and *Escherichia coli* was demonstrated for films containing 7 or 10 wt% of PHMG-NS, through a local biocide effect possibly based on an influence on the bacterial eDNA. The biocide hardly leached from the PA-11 matrix into water, at a rate of less than 1% from its total content for 21 days.

## 1. Introduction

Produced from a renewable source, polyamide 11 (PA-11) is a unique thermoplastic polymer with excellent functional properties, combining high ductility and mechanical strength, dimensional stability, low density, excellent abrasion and fatigue resistance, a low friction coefficient, high barrier properties and resistance to many types of chemicals. Many industries around the world (automotive, transport, textile, oil and gas, wire and cables, and electronics) have been using PA-11 for decades [1]. PA-11 powder coatings were developed to protect metal parts from corrosion, particularly for the protection of steel in the fluid transfer industry, for example in pipes and fittings in water treatment plants, water/hydrocarbon transportation pipelines, transport and building constructions, medical equipment, office furniture, and many other appliances [2].

It is known that the surface of polyamide plastics can be colonised rapidly by bacteria, fungi and algae, especially in a humid environment, which is then followed by the formation of biofilms/fouling. Moreover, the emerging biofilm causes contamination, staining, odours and eventually deterioration of the mechanical properties of the plastic because of the degradation of the polymer, utilising the carbon in the course of development as a nutrient [3,4,5]. Biofilm formation starts with the deposition of different microorganisms on the surface of the material, followed by growth and spreading of the colonies forming a highly complex structure, culminating in microbial evolution and adaptation towards a stronger resistance to antibiotics and biocides, the appearance of super-biofilm with super-mucous and super-adhesive opportunistic strains, etc. [6,7,8,9,10]. Bacteria foul medical devices and implants, e.g., polymeric materials used as internal or invasive devices such as catheters, components of cardiac pacemakers, artificial heart valves and joints. The formed biofilm can initiate degradation of the material, as well as hospital-acquired infections, for example of small medical devices, because of a high concentration of microorganisms. These implant-associated biofilms are often difficult to remove, even after cleaning the implants pre-operatively with oxidisers and detergents or treating them with antibiotics, and in certain situations replacement surgery may be required [6].

The introduction of antimicrobial agents into the base polymer of these articles is considered the most efficient approach to prevent the growth of biofilms on their surface. A wide variety of organic and inorganic biocides is available, whether synthetic or nature-inspired [11,12,13,14,15]. The choice of appropriate biocides for PA-11 is strongly limited because of the high processing temperatures of the polymer, up to 300 °C. Currently, silver-based compounds are the most widely used antimicrobial additives for PA-11 because of their excellent thermal stability [16,17], as well as their low toxicity to human cells [18]. Silver nanoparticles are regarded as the most promising biocides for polyamide resins since their high surface area ensures an efficient release of Ag^+^ species into the medium [19,20,21]. However, it should be noted that silver nanoparticles are hard to disperse in a polymer matrix because of their strong aggregation ability [16,17,21]. Moreover, silver ions are known to interact with polyamides during melt processing and cause an undesirable discolouration of polymer articles. Therefore, many antimicrobial formulations contain silver-based compounds intercalated into inorganic anion exchangers or encapsulated using soluble ceramics [16,17].

Thus, there is a growing demand for the development of low-cost and low toxic antimicrobial agents that combine good compatibility and processability with polyamide resins, as well as high leaching resistance from polymer matrix.

Copper and its compounds have also emerged as promising antimicrobial additives for polyamides, being much cheaper than silver. Thus, a commercially available ionic copper-based additive, Plasticopper, was incorporated into the PA-11 matrix during the polymer processing stage [22]. The incorporation of 5% and 10% copper was found to have a reinforcing effect on the composites and did not adversely affect their mechanical performance. These composite systems showed long-term antimicrobial activity against Gram-negative bacteria (*E. coli*) with a reduction of the bacterial population of more than 99.99% [22].

Nowadays, cationic polymers are being considered as a new generation of biocides because of their enhanced antimicrobial activity, as well as their low toxicity to human cells, compared to common low molecular cationic surfactants [23,24]. In particular, polyhexamethylene guanidine (PHMG) salts, comprising guanidinium cations in the main chain, are receiving increasing attention since they display a broad range of antimicrobial activity against bacteria, fungi and viruses [25,26,27,28,29,30,31], as well as antifouling activity against macrofoulers in an aquatic environment [32,33]. It should also be noted that PHMG salts showed a much lower acute toxicity than copper-based biocides [33]. The high activity of guanidinium-based polymeric biocides against microorganisms is caused by the presence of multiple positive charges within a single molecule that are able to compensate the negative charges present on the outer cell membranes of microbes. Because of these strong electrostatic interactions, PHMG is able to attack the cellular envelope, and subsequently associates itself with the head groups of the acidic phospholipids. The presence of hydrophobic aliphatic chains in the PHMG backbone ensures a better partition to the hydrophobic regions of the phospholipid membrane, resulting in a change of membrane permeability and lethal leakage of cytoplasmic materials [27,28,29,30,31].

Poorly water-soluble PHMG salts such as PHMG stearate, PHMG sulfanilate, or PHMG dodecylbenzenesulfonate (PHMG-DBS) have been reported as efficient antimicrobial additives for polycaprolactone [34], polylactide [35], polyamides [36,37] and silicones [38]. PHMG-DBS was found to have sufficient thermal stability to be melt processed with PA-11 and PA-12 resins by conventional methods [36,37]. PA-11 films containing 5–7 wt% of PHMG-DBS showed a high activity against *E. coli*, as well as an excellent resistance against leaching of the polymeric biocide [37].

Another successful approach involves the intercalation of the cationic polymer (partially aminated poly(vinylbenzyl chloride)) into a smectic clay, montmorillonite, to produce a modified organoclay containing 33 wt% of polymeric biocide [39]. Polymerically modified organoclay was found to have sufficient thermal stability for joint processing with PA-6 resin by melt extrusion. The obtained nanocomposites were active against both Gram-negative *E. coli* and Gram-positive *S. aureus* bacteria and demonstrated up to a 2-log reduction in the viable cells adhering to the material surface at an organoclay content of 5 wt%. The mode of antimicrobial action of this material was determined as contact-active because the biocide does not leach out [39].

Despite the evidence for a pronounced antibacterial and antifungal activity of contact-active polyamide composites, their activity against biofilms has not yet been studied. It is worth noting that antibiofilm characteristics do not always parallel antibacterial characteristics. There are several examples of compounds that were added to polymers and possessed antibacterial characteristics, but could not decrease fouling, and vice versa [40,41,42]. In the present study, a new thermally stable hydrophobic cationic polymer, polyhexamethylene guanidine 2-naphtalene sulfonate (PHMG-NS), was synthesised. In contrast to the previously reported polymeric biocide PHMG-DBS, which sticks together during storage, PHMG-NS forms a fine powder that makes it a good candidate to be applied in PA-11 based powder coating formulations. The aim of our research was to investigate the antibiofilm activity of PA-11 films modified with PHMG-NS biocide against the biofilm-forming model bacterial strains, opportunistically pathogenic *E. coli* K12 and *S. aureus* ATCC 25923.

## 2. Results and Discussion

### 2.1. FTIR Analysis of PA-11/PHMG-NS Films

**Figure 1:**
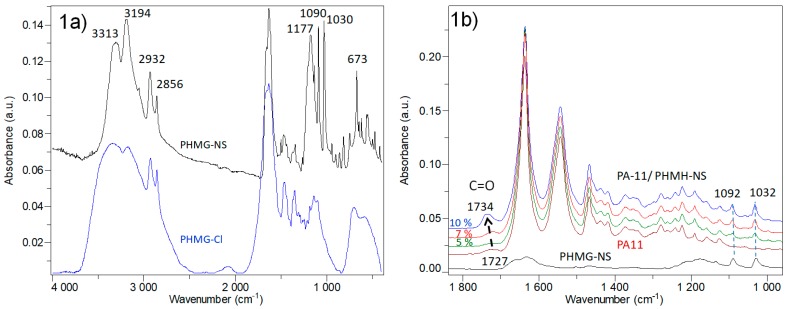
IR spectra: (**a**) PHMG-Cl and PHMG-NS; and (**b**) PHMG-NS, PA-11 and PA-11/PHMG-NS film with 5%, 7% and 10% of PHMG-NS.

Infrared spectroscopy was employed both to identify the presence of PHMG-NS inside the polymer films and reveal potential interactions between PA-11 and the biocide. Figure 1a shows infrared spectra of PHMG-Cl and PHMG-NS in the 400–4000 cm^−1^ region and Figure 1b shows infrared spectra in the 960–1830 cm^−1^ region of PA-11 and PA-11/PHMG-NS films containing 5%, 7% and 10% of PHMG-NS. As previously described for PHMG-Cl, which was the precursor compound for making PHMG-NS, the very broad bands in the region of 2600–3700 cm^−1^ are attributed to the CH_2_, NH_2_, and OH stretching vibrations [33]. The modification of PHMG-Cl to PHMG-NS does not change the position of vibrational modes, whose maxima are found at approximately 2856 and 2932 cm^−1^, respectively, and attributed to the symmetric ν_s_(CH_2_) and asymmetric ν_as_(CH_2_) stretching vibrations of the methylene groups. However, the bands attributed to the symmetric ν_s_(NH_2_) and asymmetric ν_as_(NH_2_) stretching vibrations of amine groups shift to higher wavenumbers at about 3194 and 3313 cm^−1^. The IR spectra show also strong absorption bands between 1500 and 1800 cm^−1^, characteristic of both the C=N stretching and the NH_2_ scissoring modes. The central position of these broad bands does not shift between PHMG-Cl and PHMG-NS. The strong absorption bands observed at 1177, 1090, 1030 and 673 cm^−1^ can be assigned to the sulphonic group of the 2-naphthalenesulfonate. Two of these later vibrational modes can easily be observed at 1092 cm^−1^ and 1032 cm^−1^ in Figure 1b (dashed lines) with PA-11/PHMG-NS films containing 5%, 7% and 10% of PHMG-NS, thus confirming the presence of the biocide in the films. Overall, the IR spectra of the modified films are similar to each other revealing that the introduction of PHMG-NS does not significantly influence the vibrational bands of PA-11. However, a noticeable change can be observed in Figure 1b: the small intensity band assigned to the carbonyl (C=O) stretching vibrations for PA-11 (at 1727 cm^−1^) shifts toward higher wavenumbers as a function of the PHMG-NS content and reaches 1734 cm^−1^ for films containing 10% of PHMG-NS (Figure 1b, black arrow). This feature suggests a modification of the specific environment of the C=O group. The guanidinium cation is known to participate in direct hydrogen bonding with backbone carbonyl groups of proteins [43], which suggests a similar interaction between the guanidinium cations of PHMG-NS and the amide carbonyl groups of PA-11.

### 2.2. Surface Properties of PA-11/PHMG-NS Films

According to the contact angle measurements data, an introduction of 5 wt% of PHMG-NS into a PA-11 film led to an enhanced hydrophilicity of the surface, which can be attributed to the increase of the polar functional groups on the polyamide surface. However, further increase of PHMG-NS content had little impact on the contact angle value (Table 1). The results of the electrophysical study of the PA-11/PHMG-NS films indicate a sharp increase of the positive surface charge density at a PHMG-NS content of 7 wt% and more (Table 1). Probably, it can be explained by the formation of an uncompensated positive charge of the guanidinium cations at the polymer surface because of specific interactions between the hydrophobic fragments of the PHMG-NS and the PA-11 matrix. It should be noted that both the improved hydrophilicity and the positive charge of the polymer surface are considered to be important factors that determine the antimicrobial efficacy [44,45,46].

### 2.3. Mechanical and Thermal Properties of PA-11/PHMG-NS Composites

It has been established that inorganic biocides have a reinforcing effect on the polyamide matrix caused by the formation of strong interfacial interactions, leading to a reduction in the mobility of polymer chains [22,39]. For example, heterogeneous PA-11/Cu antimicrobial nanocomposites showed an increase of the yield strength from 45 to 54 MPa when 2–10% of a copper additive was introduced [22]. The introduction of 5% of cationic biocide intercalated organoclay into PA-6 improved the yield strength by 44.6% [39].

The polymeric biocide PHMG-NS forms homogeneous composites with PA-11 because of its low melting temperature, as well as its good compatibility with the polymer matrix. Tensile testing of PA-11/PHMG-NS samples was performed to evaluate the effect of the antimicrobial additive on the mechanical properties of polyamide. PA-11 films containing 5% and 7% of PHMG-NS have tensile strength values similar to pure polyamide. A further increase of the polymeric biocide content to 10% led to a deterioration of the mechanical properties of the material (Table 2).

**Figure 2:**
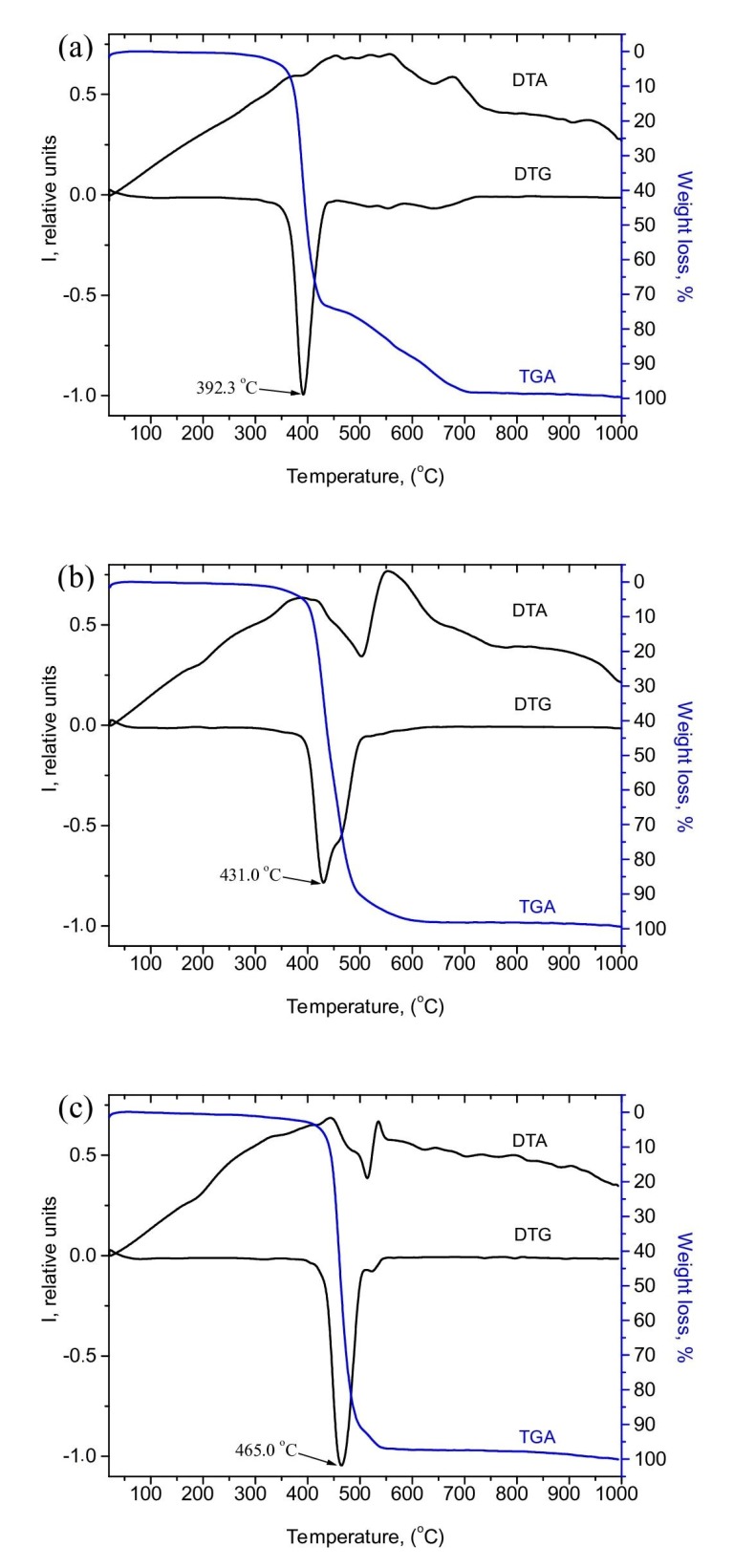
TGA curves in air of: PHMG-NS (**a**); PA-11 (**b**); and PA-11/PHMH-NS (10%) (**c**).

The results of the thermal characterisation of the polymeric biocide PHMG-NS, PA-11 and PA-11/PHMG-NS containing 10 wt% of polymeric biocide are summarised in Figure 2 and Table 3. According to the TGA data, PHMG-NS has a thermal decomposition point (which was defined as the temperature of 5% weight loss (T_Δm_ = 5%) at 357 °C. The peak mass loss temperature of PHMG-NS is 392 °C (Figure 2a). Pure PA-11 begins to decompose at 425 °C and the maximum rate of thermal degradation was observed at 431 °C (Figure 2b). The PA-11/PHMG-NS composite containing 10 wt% of PHMG-NS is thermally stable to at least 391 °C, and the peak mass loss was found at 465 °C (Figure 2c). At a lower PHMG-NS content, the thermal stability of a composite becomes closer to pure PA-11 (Table 3). Hence, PHMG-NS has excellent thermal stability and can be used for joint melt processing with polyamide resins by conventional methods, as previously reported for the polymeric biocide PHMG-DBS [36,37]. However, PHMG-NS seems to have a broader application range, due to its hydrophobic powder state, whereas PHMG-DBS has a tendency for aggregation during storage. These properties of PHMG-NS allow for its introduction into PA-11-based powder coatings for covering metal articles, by conventional methods using a fluidised bed method of air suspension of a composite material or an electrostatic spraying process.

### 2.4. Antibiofilm Efficacy of PA-11/PHMG-NS Films

**Figure 3:**
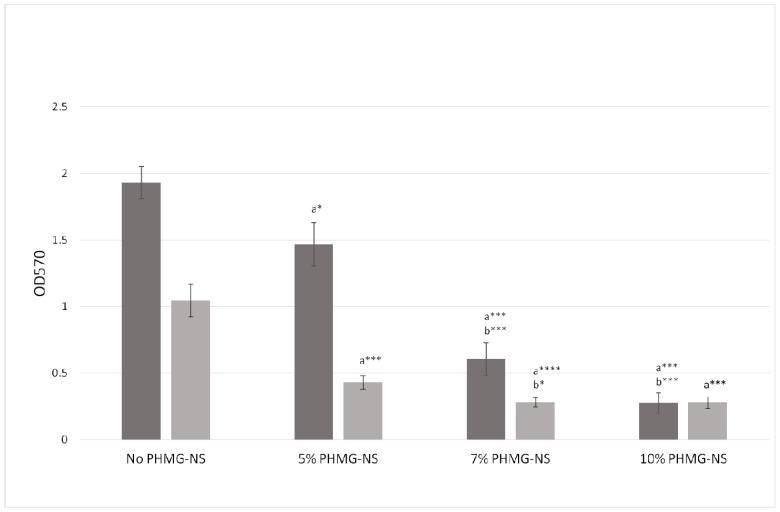
The level of biomass of *E. coli* K12 (dark grey) and *S. aureus* ATCC 25923 (light grey) biofilms formed onto PA-11 films containing 5%, 7% and 10% of PHMG-NS following 72 h of incubation determined by Crystal Violet staining and measured as the optical density at 570 nm (OD570). a: statistical significance compared to control (No PHMG-NS); b: statistical significance compared to 5% PHMG-NS. * *p* < 0.05, *** *p* < 0.005, **** *p* < 0.001.

The antibiofilm/antifouling properties of PA-11 modified with the polymeric biocide PHMG-NS were evaluated using two opportunistic biofilm-forming model strains: the Gram-negative *E. coli* K12 belonging to the Enterobacteriaceae family [47] and the Gram-positive *S. aureus* ATCC 25923 [48]. Overall, *E. coli* has been shown to be more resistant to PHMG-containing biocides than the Gram-positive opportunists, such as methicillin-resistant *Staphylococcus aureus* [34,35,38,49]. In addition, its enzymes are more stable when in contact with PHMG derivatives [35]. Principally, both strains were able to form solid–liquid biofilms to the PA-11 films within three days of cultivation. The level of biofilm biomass attached to a PA-11 film containing 5%, 7% or 10% of PHMG-NS was found to be significantly different from those on the control PA-11 films in the Crystal Violet assay (Figure 3) [50]. The biofilm formation decreased approximately three times for the samples containing 7% and 10% of PHMG-NS. 

**Figure 4:**
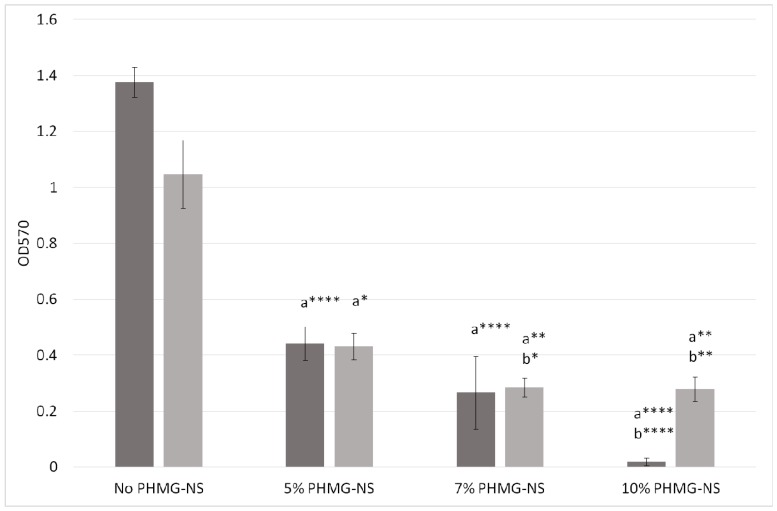
The level of metabolic activity of *E. coli* K12 (dark grey) and *S. aureus* ATCC 25923 (light grey) biofilms formed onto PA-11 films containing 5%, 7% and 10% of PHMG-NS following 72 h of incubation determined by MTT staining and measured as the optical density at 570 nm (OD570). a: statistical significance compared to control (No PHMG-NS); b: statistical significance compared to 5% PHMG-NS. * *p* < 0.05, ** *p* < 0.01, **** *p* < 0.001.

The Crystal Violet assay measures the overall level of organic layers formed on a plastic surface, containing bacterial cells and numerous organic molecules integrated into the biofilm matrix. Of course, contact-active antimicrobial surfaces are often coated with a layer of dead microbes, to which newly approaching microbes can also adhere and proliferate [51]. Hence, a higher level of Crystal Violet staining does not always correspond to a higher metabolic activity of a biofilm. To assess whether the metabolic activity of the biofilm could be related to the overall number of living bacterial cells in the biofilms, an MTT test was performed in parallel [52]. In this assay, a similar tendency was revealed. All PHMG-NS-containing PA-11 films showed a significant reduction in biofilm metabolic activity (Figure 4). The level of biofilm metabolic activity was 2.5 times lower on the PA-11/PHMG-NS (5%) than on the control PA-11 films. There was a significant difference between the growth under control conditions and the growth on PA-11 containing 5%, 7% and 10% of the polymeric biocide. There was no significant difference between samples containing 5% and 7% PHMG-NS, and between PA-11/PHMG-NS (7%) and PA-11/PHMG-NS (10%). Generally, there was a fivefold decrease in the biofilm metabolic activity for polymer films containing 7% PHMG-NS compared to the control PA-11 films for both bacterial strains (*p* < 0.001 for *E. coli* and *p* < 0.01 for *S. aureus*), and there was almost no metabolic activity on films with 10% PHMG-NS inoculated with *E. coli*.

**Figure 5:**
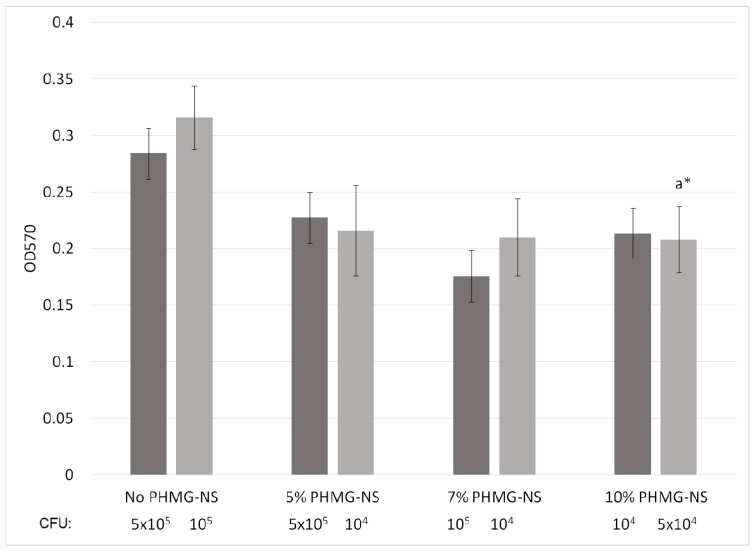
The level of planktonic cell biomass (overbiofilm layer indicated by the optical density at 570 nm (OD570)) of K12 (dark grey) and ATCC 25923 (light grey) in the presence of PA-11 films containing 5%, 7% and 10% of PHMG-NS following 72 h of incubation, correlated with the corresponding CFU counts. a: statistical significance compared to control (No PHMG-NS); * *p* < 0.05.

The bacterial toxicity of the PA-11/PHMG-NS films for the planktonic part of the bacterial culture was evaluated by measuring both the optical density and the colony forming units (CFU) count in the overbiofilm layer in 72-h stationary biofilm-forming K12 and ATCC 25923 cultures. There was no significant difference in optical density between any of the four samples (Figure 5) except a minor and possible negligible decrease of ATCC 25923 in the presence of PA-11/PHMG-NS (10%) (*p* < 0.05). The planktonic CFU numbers in the media in contact with the control and 5% PHMG-NS films were, respectively, tenfold and fivefold higher than the CFU of the plankton in contact with the PA-11/PHMG-NS (10%) and the PA-11/PHMG-NS (7%) films for K12. There was also a tenfold decrease in plankton density in PA-11 films doped with 5% and 7% of PHMG-NS and a fivefold decrease for PA-11/PHMG-NS (10%) compared to control PA-11 for ATCC 25923. However, there was no significant difference in effect between the different polyamide films on the planktonic bacteria in a biofilm-forming culture, suggesting a low release rate of the biocide into the medium in the experimental conditions presented here. 

The behaviour of the planktonic part of the culture raised a question about the antibiofilm properties of the PA-11 films after water exposure. To investigate this, pieces of polymer containing 7% and 10% of PHMG-NS were exposed to water for seven days. The biofilm assay did not show any significantly different antibiofilm properties between the exposed and the non-exposed films. 

**Figure 6:**
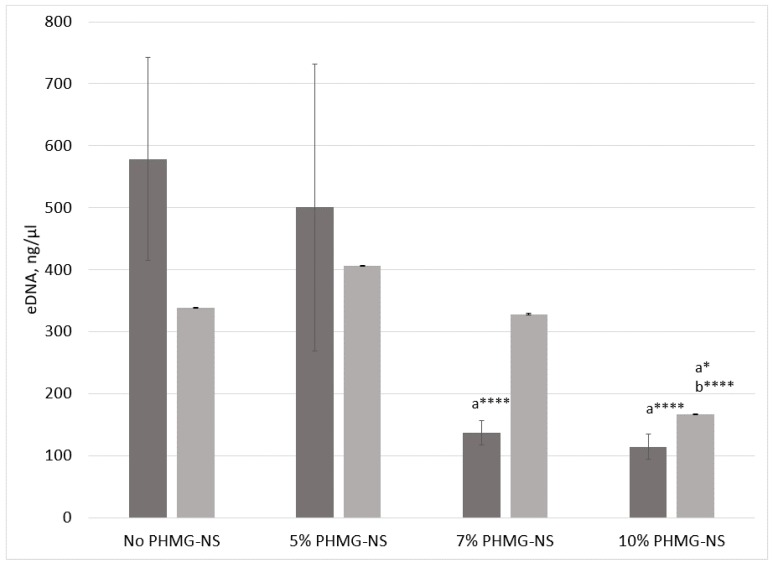
Amount of eDNA precipitated from a biofilm layer (solid-bottom phase) of 72-h-old biofilm-formed K12 (dark grey) and ATCC 25923 (light grey) cultures with PA-11 films containing 5%, 7% and 10% of PHMG-NS. a, statistical significance compared to control (No PHMG-NS); b: statistical significance compared to 5% PHMG-NS. * *p* < 0.05 **** *p* < 0.001.

**Figure 7:**
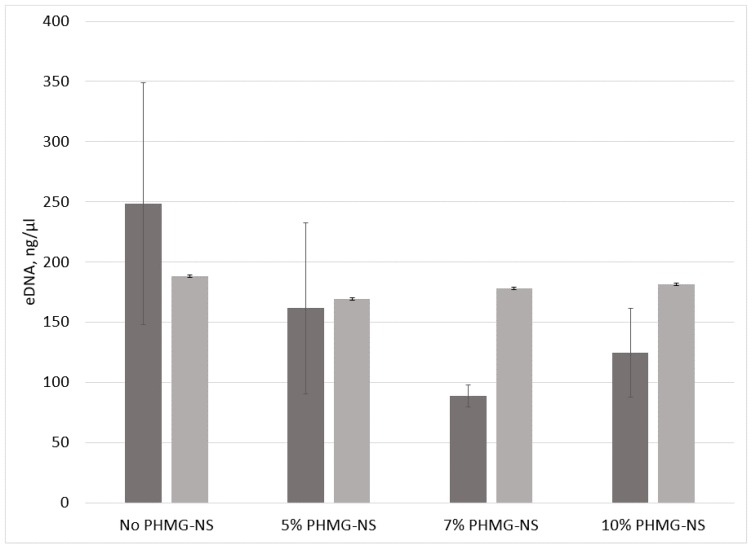
Amount of eDNA precipitated from overbiofilm layer (liquid plankton phase) of 72-h-old biofilms-formed K12 (dark grey) and ATCC 25923 (light grey) cultures with PA-11 films containing 5%, 7% and 10% of PHMG-NS.

Despite the absence of any significant effect of the presence of PHMG-NS in the PA-11 on the growth of planktonic cells, visual observation of the 72-h cultures revealed that the biofilm formation on the solid–liquid interface of the microcosms was unexpectedly reduced by the presence of at least 7% of PHMG-NS in the PA-11 films (direct observations). This raises a question: is there any distant effect of non-leaching PHMG-NS onto biofilm formation in a microcosm? Biofilms consist of bacterial cells embedded into a matrix of extracellular polymers composed of polysaccharides, proteins and nucleic acids (mostly DNA) [53]. Extracellular DNA (eDNA) has been known to play quite an important role in the initiation and the development of biofilms of many bacteria [54,55,56,57], among which those made by *E. coli* and *S. aureus* [58,59]. eDNA is a negatively charged molecule which might interact with the positively-charged PHMG-NS-containing PA-11. To check this hypothesis, the total eDNA content in the culture was precipitated from the solid–liquid interface biofilms formed after 72 h in the microcosms (Figure 6). There was a significant decrease in eDNA content in the biofilms formed in the presence of PA-11/PHMG-NS (7%) and PA-11/PHMG-NS (10%) for K12 (*p* < 0.001) and in the presence of only PA-11/PHMG-NS (10%) for ATCC 25923 (*p* < 0.05 compared to the control culture and *p* < 0.001 compared to the 5% PHMG-NS treatment), each time compared to the eDNA amount found in microcosms exposed to control PA-11 and PA-11/PHMG-NS (5%), while there were no such differences in the overbiofilm layer in any of the microcosms (Figure 7). Such a decrease in eDNA in the solid–liquid interface biofilms, in contrast with the constant level in the overbiofilm culture, might suggest that at least one of the antibiofilm mechanisms of PHMG-NS-containing PA-11 is associated with eDNA reduction, which in turn may have an influence on the initial stages of bacterial biofilm formation. Moreover, decreasing the level of eDNA would hypothetically reduce an abundance of resistance genes spread horizontally in a hospital-related environment, as eDNA associated with biofilms has been considered a hotspot for deposition and recombination of antibiotic resistance genes [60,61].

It is worth noting that the reported antimicrobial polyamide composites have never before been studied for their activity against biofilms. However, polylactide films containing hydrophobic PHMG salts were found to strongly inhibit the activity of bacterial intracellular dehydrogenases, which prevented the formation of microbial biofilms on the polymer surface [35].

### 2.5. Leaching Resistance of PHMG-NS from PA-11 Films

**Figure 8:**
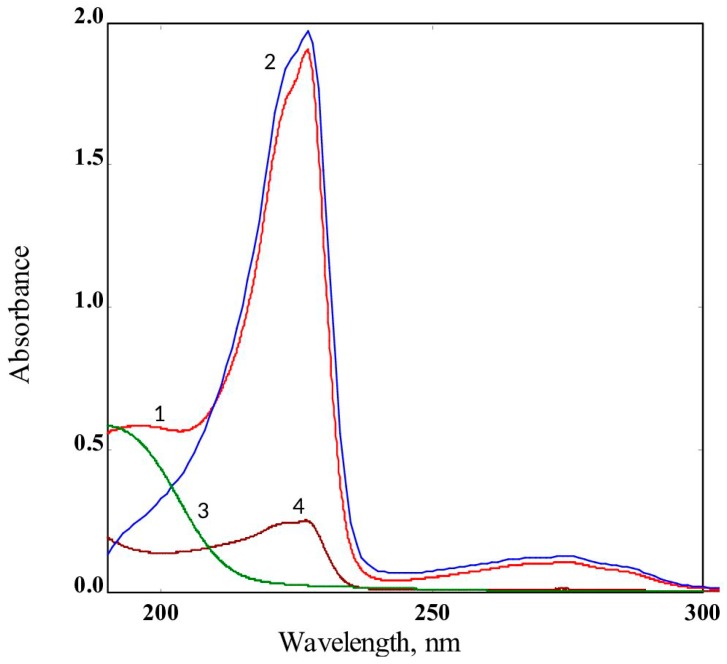
UV-visible spectra of: PHMG-NS (1); sodium 2-naphthalene sulfonate (2); PHMG hydrochloride (3) (C = 4.5 × 10^−5^ mol/L), and water solution after seven-day contact with PA-11/PHMG-NS (7%) film (4).

The water solubility of PHMG-NS was found to be 0.24 g/L. Thus, given the possibility of a non-covalent association between the PA-11 matrix and PHMG-NS, a gradual release of polymeric biocide into the aqueous medium could be expected. Figure 8 contains UV-visible spectra of PHMG-NS (Curve 1), as well as its precursor PHMG-Cl (Curve 3), which is highly soluble in water. As one can see from these spectra, the characteristic ultraviolet absorption of the guanidyl carbon-to-nitrogen double bond of PHMG allows for a spectrophotometric analysis of either compound in aqueous solutions [62]. However, the adsorption peak of the 2-naphthalene sulfonate anion at 227 nm is the most expressive (Figure 8, Curve 2) and therefore was used for PGMG-NS detection.

After three days of contact with warm water, the PA-11/PHMG-NS (7%) film had lost less than 1% of its biocide contents (Figure 8, Curve 4). No further release of biocide was detected after 7, 14 and 21 days of exposure, which indicates that the biocide-doped polymer is highly resistant to leaching, which in turn is important for the potential durability of the antimicrobial activity of the material. It is worth noting that PA-11 has a much lower water absorption than other commercial polyamides [1], which may be a crucial factor in the determination of low biocide release rate [19]. Moreover, the cooperative hydrogen bonding of both guanidinium cations and 2-naphthalenesulfonate anions with the polar amide groups may also ensure the high retention of PHMG-NS in PA-11. 

Even though the non-covalent association between the bioactive compounds and the polymer matrix has been a major advantage to optimise the antimicrobial performance [24,51], the question always remains: is the system contact-active or biocide-releasing? It was shown for antimicrobial polyamide nanocomposites containing silver- or copper-based inorganic biocides that they release Ag or Cu ions into the surrounding aqueous medium in a steady and prolonged manner [19,20,21,22]. At the same time, biocide release was detected neither for polylactides, polyamides or silicones containing hydrophobic PHMG salts [35,36,37,38] nor for PA-6 containing cationic biocide-modified organoclays [39]. Thus, the mode of antimicrobial action of these materials can be determined as contact-active because of the non-leachable form of the biocide. Bacterial membranes are known to carry a large number of negative charges and therefore can adsorb on positively charged polymer surfaces [24,46,63]. Upon adsorption on a cationic solid substrate, the electrostatic compensation of the negative charges of the bacterial envelope is provided by the cationic charges of the substrate, and the bacteria lose their natural counterions. It has been suggested that this counterion release initiates bacterial death. In the case of Gram-negative bacteria such as *E. coli*, Mg^2+^ and Ca^2+^ ions, which stabilise the outer membrane of the bacterial cell, are expelled during the adsorption of the bacteria on the charged substrate. Thus, the outer membrane is destabilised, leading to non-viable cells [24].

In our study, the low antimicrobial activity of PA-11/PHMG-NS films against planktonic bacteria may indicate that its antimicrobial action is based on contact-killing. The negligible release of polymeric biocide from polyamide matrix may also testify in favour of this assumption. As mentioned above, the antibiofilm properties of both PA-11/PHMG-NS (7%) and (10%) films were not altered after a seven-day water exposure, suggesting that the contact-killing mode of action as well as the anti-eDNA effect were the main elements in the antibiofilm/antifouling mechanism.

## 3. Materials and Methods

### 3.1. Materials

The following chemicals were used without further purification for the synthesis of the polymeric biocide: guanidine hydrochloride, 98% (Applichem, Darmstadt, Germany), hexamethylenediamine (98%), 2-naphtalenesulphonic acid, sodium salt (technical grade, Sigma-Aldrich, Taufkirchen, Germany), and ethanol (95%). Rilsan^®^PA11 (granules) was supplied by Arkema (King of Prussia, PA, USA).

### 3.2. Synthesis of Polymeric Biocide PHMG-NS

**Scheme 1:**
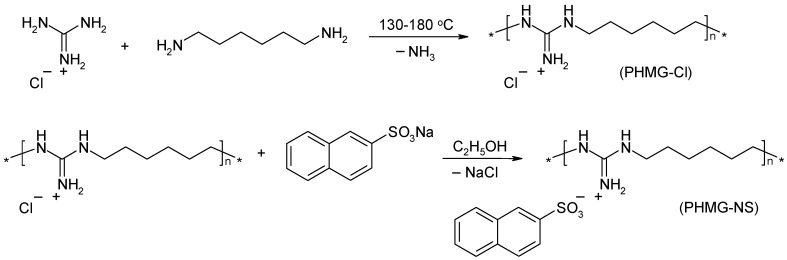
Synthesis of polymeric biocide PHMG-NS.

PHMG-NS was synthesised according to the following procedure (Scheme 1).

A mixture of guanidine hydrochloride (10 g, 0.104 mol) and hexamethylenediamine (12 g, 0.103 mol) was placed into a round-bottomed flask (250 mL) and heated at 80 °C for 4 h under constant stirring. Subsequently, the reaction was carried out for 5 h at 130–140 °C and 5 h at 180 °C to obtain a highly viscous melt. After cooling the reaction mixture to room temperature, a vitreous solid PHMG-Cl was obtained. It was dissolved in water (200 mL) and precipitated by adding 100 mL of a saturated sodium chloride solution. The polymer was isolated by decantation and dried at 140 °C for 24 h. The product yield was 13 g (72%). The intrinsic viscosity was 0.09 dL/g for a PHMG-Cl solution in 0.1 N NaCl at 25 °C.

Sodium 2-naphtalenesulfonate (13.6 g, 0.06 mol) was added to the solution of PHMG-Cl (10 g, 0.055 mol) in 200 mL of ethanol and the mixture was stirred for 12 h at 60 °C (Scheme 1). The formed sodium chloride precipitate was filtered off and the solution was poured into water (500 mL). The white slurry of PHMG-NS was separated by the decantation and washed with water. The wet product was dried at 130 °C for 24 h and then powdered in an agate mortar. The product yield was 16 g (87%). The PHMG-NS salt has a melting point of 105–110 °C. Its water solubility was found to be 0.24 g/L.

The ^1^H NMR and elemental analysis data for PHMG-Cl and PHMG-DBS are shown in the Supplementary Materials.

### 3.3. Preparation of PA-11/PHMG-NS Composite Films

PA-11 granules were dissolved in formic acid (98%) at 50 °C to obtain a 10 wt%. solution. An equal volume of isopropanol was added dropwise to the stirred solution to precipitate the polyamide powder. It was filtered off and then washed successively with sodium hydroxide (5%) and water. The obtained fine powder was dried in vacuum (1 mbar) at 70 °C for 12 h.

The mixture of PA-11 and PHMG-NS powders was ground for 3 min in an agate mortar followed by compression moulding at 240 °C. Composite polyamide films (45 mm × 45 mm) were obtained containing 5, 7 and 10 wt% of polymeric biocide.

### 3.4. Characterisation of PA-11/PHMG-NS Composite

To characterise the chemical properties of the modified PA-11 films, the samples were first placed on the Platinum diamond ATR module and IR spectra were recorded using a Bruker Vertex-70V FTIR spectrometer (all Bruker Optics Inc., Ettlingen, Germany) equipped with a L-alanine-doped deuterated triglycine sulphate (DLaDTGS) detector. Spectra were acquired with a resolution of 2 cm^−1^ in the spectral region from 400 to 5000 cm^–1^ as the co-addition of 100 scans. Acquisition of these spectra was done with Bruker OPUS software (version 6.5, Ettlingen, Germany).

Mechanical testing of the polyamide samples was performed using a P-50 universal tensile testing machine (Milaform, Moscow, Russia) at a deformation rate of 10 mm/min. The obtained films were cut into specimens with the size of 40 mm × 10 mm × 0.15 mm. An average value (with standard deviation) for the tensile strength was obtained from three samples of each film.

Contact angle measurements were performed using a Drop Shape Analyzer DSA25E (Krüss, Hamburg, Germany) by the sessile drop method. The contact angle was estimated, using ImageJ software (version 1.50i, Bethesda, MD, USA), as the tangent normal to the water drop (3 mL) at the intersection between the sessile drop and the polymer surface. All reported contact angles are the average of at least five measurements taken at different locations on the polymer surface.

The surface charge density of the PA-11/PHMG-NS films was determined with the contactless inductive method [37]. The surface charge of the samples was determined by comparing the voltage amplitudes of the capacitor with PA-11 films and of the capacitor with a known calibrated electret. The measurements were carried out immediately after the samples were positioned into the measuring assembly, as well as 5 min later to estimate the changes of the surface charge with time.

Thermal gravimetric analysis (TGA) data for the polymeric biocide and modified PA-11 samples were obtained using a TGA Q500 (TA Instruments, Eschborn, Germany). About 10 mg of each sample were heated from 30 °C to 700 °C with a heating rate of 10 °C/min under an air atmosphere.

### 3.5. Biocide Release

The solubility of pure PHMG-NS in water was determined by stirring 1 g of the polymeric biocide powder in 100 mL of water for 24 h at room temperature. Then, the solution was filtered and evaporated. The weight of the solid residue was determined.

The release of PHMG-NS from the PA-11 film was investigated by UV-visible spectrophotometric analysis using a Jenway 6850 spectrometer (Stone, United Kingdom). The calibrating graph was obtained by measuring the absorbance of PHMG-NS aqueous solutions in a concentration range of 1 × 10^−5^–5 × 10^−5^ mol/L at 227 nm, which is the characteristic peak of the naphthalene ring. For the evaluation of the leaching rate of polymeric biocide, 2 g of PA-11/PHMG-NS (7%) film was placed into a closed 1 L conical flask containing 750 mL of deionised water. The sample was kept at 37 °C at constant stirring. Three millilitres of each solution were taken periodically and analysed by measuring the absorbance at said wavelength to determine the concentration of the released biocide. The biocide release ratio was determined as the percentage of PHMG-NS released into the solution from its total quantity in the film. Each measurement has been repeated three times.

### 3.6. Biofilm Assay

The resistance to biofouling, i.e., the antibiofilm characteristics of the PA-11/PHMG-NS films, was evaluated by assessing the capability of two biofilm-forming model strains, *E. coli* K12 and *Staphylococcus aureus* ATCC 25923, to form attached biofilms on the surface of polymer samples following three days of stationary incubation. Each PA-11/PHMG-NS film was cut into pieces of 1 cm^2^ each. The pieces were sterilised by autoclaving at 105 °C for 30 min. Water-treated pieces of plastics were prepared by exposition of 1 g of plastic films in 500 mL of deionised water for 7 days.

Each piece was then placed in a well of a sterile polystyrene 24-well plate in which 2 mL of Luria Broth (LB) medium were added, inoculated with 10 µL of an overnight inoculum culture containing 10^9^ CFU/mL; there were eight replicas per variant. The plate was incubated at 37 °C for 72 h. The control for incubation was performed by incubating the films in sterile LB with four replicas. After incubation, each film piece was removed and washed three times to remove planktonic and poorly attached biofilm mass. 

To measure the level of biofilm biomass attached to the plastics, a Crystal Violet assay was performed. To this end, eight pieces with attached biofilms were placed in a glass vial, stained with 1 mL of a 0.05% Crystal Violet stain solution for 30 min, and washed three times in water, after which the stain was eluted by incubating the piece in 500 µL of 96% ethanol for an hour. The eluted stain in each 200 µL aliquot was quantified by absorbance measurements at 570 nm in a BioTek ELx800 microplate spectrophotometer. Net biofilm attachment was calculated by subtracting the control values, corresponding to an incubation in LB.

A biofilm metabolic assay was performed for eight pieces as well. For this, each piece was placed in a glass vial with 500 µL of a 0.05% methylthiazolyldiphenyltetrazolium bromide (MTT) solution (Sigma-Aldrich, Taufkirchen, Germany) and incubated at 37 °C for 20 h. Then, the film and the MTT solution were removed from each vial and placed in a 1.5 mL plastic tube, which was spun down at 13,000× *g* for 15 min (Eppendorf 5424 Microcentrifuge (Fisher Scientific, Pittsburg, PA, USA). The supernatant was discarded and the sediment was dissolved again in 500 µL of DMSO. Again, staining intensities of 200 µL were evaluated using absorbance measurements at 570 nm. Net biofilm metabolic activity was calculated by subtracting the control values, corresponding to an incubation in LB.

### 3.7. Bacteria Toxicity Assay

The toxicity of the PA-11/PHMG-NS films was assessed on the basis of the optical density as well as the colony forming units number of the overbiofilm planktonic culture. For this, 200 µL of the overbiofilm planktonic culture of each treatment were transferred into a sterile polystyrene 96-well plate. The optical density was measured spectrophotometrically at 570 nm [64]. One hundred microlitres of each overbiofilm planktonic culture were used for preparing dilution series which were subsequently plated on LB agar plates for a colony forming units determination.

### 3.8. eDNA Assay

The amount of extracellular DNA (eDNA) was measured after its precipitation from cell-free supernatants. For this, 500 µL of the aforementioned biofilm culture and 550 µL of the bottom culture containing solid–liquid interface biofilms of the same microcosm, in which the PA-11/PHMG-NS films had been incubated for 72 h, were vortexed for 1 min and spun down at 13,000× *g* for 15 min (Eppendorf 5424 Microcentrifuge). Five hundred microlitres of each supernatant were removed and transferred into new 1.5 mL plastic tubes. One millilitre of chilled 96% ethanol and 50 µL of a 3 M sodium acetate solution (pH 5.2) were added to the supernatants, which were then incubated overnight at –20 °C. Then, the samples were spun down at 13,000× *g* for 15 min and the supernatants were removed. One millilitre of 70% ethanol was added into each of the tubes, which were then spun down again at 13,000× *g* for 15 min to wash the pellet. The supernatants were removed and the sediments were dried to full ethanol evaporation. Two hundred microlitres of Tris-EDTA (TE) buffer were added to dissolve each sample. eDNA concentration was measured using a NanoDrop 2000 spectrometer (Thermo Fisher Scientific, Waltham, MA, USA).

### 3.9. Statistical Analysis

The obtained data were processed statistically using the software package Statistica 7 or MS Excel for Windows. All results are presented as mean ± standard deviation. A value of *p* < 0.05 was considered statistically significant.

## 4. Conclusions

A new, thermally stable polymeric biocide polyhexamethylene guanidine 2-naphtalenesulfonate (PHMG-NS) was synthesised by anion metathesis between polyhexamethylene guanidine hydrochloride and sodium 2-naphtalenesulfonate. In dried conditions, PHMG-NS can be prepared as fine powder, which makes it suitable as an antimicrobial additive for polymer articles and protective coatings. It has a melting point of 105–110 °C and a limited water solubility of 0.024 g/L. According to thermogravimetric analysis, PHMG-NS is thermally stable to at least 357 °C, which indicates its availability for joint melt processing with polyamide resins by common methods. Polyamide 11 (PA-11) films containing 5, 7 and 10 wt% of PHMG-NS have been obtained by compression moulding at 240 °C. The introduction of PHMG-NS into PA-11 films was found to significantly increase its surface hydrophilicity, as well as positive surface charge density. PA-11/PHMG-NS composites showed no changes of tensile strength at PHMG-NS content up to 7%, as well as high thermal decomposition point about 400 °C. 

The antibiofilm properties of PA-11 modified with the polymeric biocide PHMG-NS were evaluated using the opportunistic biofilm-forming model bacterial strains *E. coli* K12 and *S. aureus* ATCC 25923. There was a substantial decrease in biofilm metabolic activity as well as in biofilm biomass for PA-11 films containing 7% and 10% of PHMG-NS for both strains. At the same time, there was no significant difference between the different PA-11/PHMG-NS films with regard to their effect on the planktonic bacteria in a biofilm-forming culture. The last fact may be due to the negligible biocide release into the medium. Indeed, the study of PHMG-NS release behaviour from the PA-11 films showed a low leaching ratio of less than 1% after 21 days, confirming its high retention in polymer matrix and maintenance its antibiofilm characteristics. Thus, it has been suggested that at least one mechanism of antibiofilm activity of PHMG-NS-containing PA-11 is associated with a reduction of eDNA, which affects the initial stages of bacterial biofilm formation and, thus, may decrease the spread of antibiotic resistance genes in a hospital-related environment.

## Figures and Tables

**Table 1:** Surface properties of PA-11/PHMG-NS films.

Sample	Contact Angle (Degree)	Surface Charge Density (C/cm^2^)
PA-11	80 ± 2	(0.47 ± 0.02) × 10^−10^
PA-11/PHMH-NS (5%)	70 ± 2	(1.2 ± 0.1) × 10^−10^
PA-11/PHMH-NS (7%)	68 ± 2	(6.5 ± 0.3) × 10^−10^
PA-11/PHMH-NS (10%)	68 ± 2	(27 ± 1) × 10^−10^

**Table 2:** Mechanical properties of PA-11/PHMG-NS composites.

Sample	Tensile Strength, MPa	Elongation at Break, %
PA-11	45 ± 2	24 ± 4
PA-11/PHMG-NS (5%)	45 ± 1	25 ± 5
PA-11/PHMG-NS (7%)	43 ± 2	15 ± 3
PA-11/PHMG-NS (10%)	27 ± 2	6 ± 2

**Table 3:** TGA data for PHMG-NS and PA-11/PHMG-NS composite*.

Sample	T_Δm_ = 5%, °C	T_Δm_ = 10%, °C	T_Δm_ = 20%, °C	T_Δm_ = 50%, °C
PHMG-NS	357	372	381	398
PA-11	425	441	450	463
PA-11/PHMG-NS (5%)	413	434	445	456
PA-11/PHMG-NS (7%)	405	430	442	449
PA-11/PHMG-NS (10%)	391	408	420	443

* Standard error *n* ± 1.

## Supplementary Materials

Supplementary materials can be found at http://www.mdpi.com/1422-0067/20/2/348/s1.

## Abbreviations

PA-11	Polyamide 11
PHMG-Cl	Polyhexamethylene guanidine hydrochloride
PHMG-NS	Polyhexamethylene guanidine 2-naphtalenesulfonate
LB	Luria Broth
MTT	Methylthiazolyldiphenyltetrazolium bromide
eDNA	Extracellular DNA
TE buffer	Tris-EDTA buffer
CFU	colony forming units
