# Supplementary material for: Antibiofilm Activity of Polyamide 11 Modified with Thermally Stable Polymeric Biocide Polyhexamethylene Guanidine 2-Naphtalenesulfonate
Source: Int J Mol Sci. 2019 Jan 16;20(2):348. doi: 10.3390/ijms20020348 (PMC6358945; doi:10.3390/ijms20020348)
Supplement: Supplementary file 1 [file ijms-20-00348-s001.pdf]

Supporting Information

^{1}H NMR and elemental analysis data for synthesized polyhexamethylene guanidine salts

PHMG-Cl

^{1}H NMR (300 MHz; DMSO-D$_6$): δ = 1.32 (m, 4H, CH$_2$), 1.47 (m, 4H, NCH$_2$C*H$_2$*), 3.16 (m, 4H, NCH$_2$), 7.1-8.1 (br s, 4H, NH).

Elemental analysis found: C: 46.4; H, 8.6; N, 24.1; Cl 20.4. Calc. for $(C_7H_{16}N_3Cl)_x$: C, 47.3; H, 9.0; N, 23.6; Cl, 20.0.

PHMG-NS

^{1}HNMR (300 MHz; DMSO-D$_6$): δ = 1.39 (m, 4H, CH$_2$), 1.21 (m, 4H, NCH$_2$C*H$_2$*), 3.07 (m, 4H, NCH$_2$), 7.18-7.46 (br s, 4H, NH), 7.54 (m, 2H, H-6, H-7), 7.75 (d, 1H, H-3), 7.88-7.97 (m, 3H, H-4, H-8, H-5), 8.19 (s, 1H, H-1).

Elemental analysis: Found, C, 56.4; H, 6.3; N, 9.9; S, 9.1. Calc. for $(C_{16}H_{23}N_3O_3S)_x$: C, 56.9; H, 6.8; N, 12.4; S, 9.5.
